# Editorial

**Published:** 2012-10-16

The current edition of the journal brings forth diagnostic and surgical techniques in glaucoma. Mingguang He et al review the clinical applications of ultrasound biomicroscopy for the anterior segment, while Sathyan et al provide insight into the applications of optical coherence tomography in glaucoma diagnosis, and Kaushik et al review the uses of ocular response analyzer in determining corneal hysteresis. Khanna and Ichhpujani detail on the use of low vision devices in glaucoma, while Khurana et al give a perspective on drug-induced glaucoma. The Ex-PRESS shunt, a new microshunt for glaucoma surgery, is presented by Angmo et al in the treatment of neovascular glaucoma. We hope that the reader will find the articles of interest and help them in improving the standard of care in glaucoma patients.

**Editors**

